# Notes from Past Meetings of the Lippe Society

**Published:** 1890-10

**Authors:** 


### NOTES FROM PAST MEETINGS OF THE LIPPE SOCIETY.

SACCHARUM-ALBUM:

Dr. Allen related a case of opacity of the cornea cured with Saccharum-album potentized. Dr. Lippe said that Bœnninghausen had proved Saccharum-album, and had shown his provings to Dr. Lippe, who copied them and afterward published them in the *Hahnemannian Monthly.* It is very similar to Calc-carb.

Dr. Allen said that he had cured swelling around the ankles following rheumatism with Saccharum-album 2M. Dr. Lippe said black-and-tan terrier dogs that eat sugar go blind.

SENSITIVENESS TO NOISE:

Dr. Lippe said that, in extreme sensitiveness to noise, the patient can hear whispers in the next room and is irritated by them; Cannabis-indica is the remedy. Nitric-acid has sensitiveness to jarring and rumbling of wagons in the street.

Dr. Lee said that Coffea has the same symptom.

Dr. Allen said that Borax has great sensitiveness to noise; to the fall of a door-latch, crumpling of paper, and rustling of silk. Coffea has general sensitiveness to all noises.

The conversation falling upon Hahnemann,

Dr. Fellger said that a new book upon Hahnemann by a Dr. Ameke had appeared at Berlin. By contemporary documents the author shows that Hahnemann was the greatest physician of his time. One of Hahnemann's peculiarities was that he got along with very little sleep. He worked prodigiously. He published one hundred and sixteen large works and about one thousand two hundred pamphlets. He was a good musician and an astronomer, and was versed in every branch of knowledge that was connected with medicine. About 1806, he remarked that Mercury had the same symptoms as syphilis, and that it would cure syphilis. When Hahnemann came out with his new system of medicine, he was universally spoken of with respect, and even reverence, but with regret for his folly. But, after a year or so, he was denounced as an ignoramus and scoundrel.

Dr. Fellger further said that this book of Ameke contains the most astonishing evidence of the meanness and rascality of the old-school physicians of Hahnemann's day.

In Hahnemann's day, it was against the law to allow a patient to die *without* bleeding him, and penalties were inflicted for neglecting to do it. Hence, there was an additional scourge in the hands of old-school doctors for punishing homœopathists.

MERCURIAL POISONING;

Dr. Fellger related a case of a man treated by an eclectic homœopathist with massive doses of Mercury. The patient died, and Dr. Fellger made a post-mortem examination. He found two ounces of metallic mercury in the liver.

He also mentioned cases where he had experimented upon patients who had taken massive doses of Mercury. He had applied the poles of a galvanic battery terminating in copper plates to the skin, and the electricity had extracted the mercury, causing it to settle upon the plates, giving them a silvery appearance.

The idea that Syphilinum is the remedy for syphilis is absurd. If so, then we have only to diagnose syphilis, and give Syphilinum to cure the case. This is pathological prescribing, and we are simply swinging around the same circle as the old school of medicine.

In the bone affections of syphilis, when there are pinkish-red swellings of the tibia—nodes—with unbearable pain, Kali-hydriodicum is the remedy. Aurum is useful for syphilitic ulcers of the roof of the mouth. Manganum-aceticum for a single small tumor of the roof of the mouth, the bone somewhat involved. Asafœtida, many small tumors of the roof of the mouth with suppuration. The tumors are discolored, this color being the distinguishing feature. The bone is deeply involved in the suppurative process.

In the case of the tibia before referred to, if the node be bluish Manganum-aceticum is the remedy. When the nodes are small and numerous Asafœtida is likely to be the remedy. Kalihydriod. is more superficial, Mangan-acet. acts more deeply. Asafœtida has a very marked action upon the periosteum.

Dr. Fellger related how a case of syphilis was caused by vaccinating with virus from a syphilitic subject.

These are but slight examples of the diverse indications that arise in syphilis, and consequently multiply the number of remedies needed to effectually treat it. Moreover, if we reflect upon the number of remedies required to treat mercurialization, which is comparatively definite in its affection, how many more must be needed to meet the requirements of so complex a disease as syphilis.

On another occasion there was a discussion upon

HYDROPHOBIA.

Dr. Lippe related a singular case which somewhat resembled hydrophobia. The patient had made her feet sore by excessive walking. So she applied turpentine, which was followed by spasms every time the patient saw water or heard it poured or saw a bright object. She also had spasms from any attempt to urinate. Cantharides relieved her.

MALARIA:

Dr. Allen spoke of a case of profuse sweat occurring in a case of malaria. The patient was confined to the bed and the sweat continued to come so freely that when the arm was held up it would run off in a perfect stream. Quinine sulphate 41M (F.) was given and the sweat abated in two days. The man then got well.

Dr. Lippe told of the case of a man whom he was treating for chills. The patient having large business interests pressing upon him, was unwilling to await the action of the similar remedy, but insisted on taking a large dose of Quinine to "break up" the chill. Dr. Lippe objected, and predicted that the patient would pay a heavy penalty by continuing to be sick for years. The patient nevertheless took the Quinine, with the result exactly as Dr. Lippe had predicted.

HEMORRHOIDS:

Dr. Guernsey had a case of a patient with hemorrhoids which protruded with sharp pain when urinating. Baryta-carb. cured.

